# The global health governance of antimicrobial effectiveness

**DOI:** 10.1186/1744-8603-2-7

**Published:** 2006-04-25

**Authors:** Greg Martin

**Affiliations:** 1 London School of Hygiene and Tropical Medicine, Keppel Street, London, WC1E 7HT, UK

## Abstract

Antimicrobial resistance is a growing threat to public health the world over. Global health governance strategies need to address the erosion of antimicrobial effectiveness on three levels. Firstly, mechanisms to provide incentives for the pharmaceutical industry to develop antimicrobials for diseases threatening the developing world need to be sought out. Secondly, responsible use of antimicrobials by both clinicians and the animal food growing industry needs to be encouraged and managed globally. And lastly, in-country and international monitoring of changes in antimicrobial effectiveness needs to be stepped up in the context of a global health governance strategy.

## 

Four and a half billion years of evolution has left the microbes that cause disease in humans, with a remarkable capacity for adaptation to changes in their micro chemical environment. This month *Globalization and Health *published a paper, "Antibiotic resistance as a global threat: Evidence from China, Kuwait and the United States" which explores the possibility of a global spread in antimicrobial resistance (AMR) and a novel technique for monitoring such a phenomenon. Whether or not AMR spread will become a global phenomenon still remains to be seen, the monitoring thereof will however be a valuable exercise, (figure 1).

The problem of AMR need to be addressed on three fronts. Firstly, on a bio-molecular level, antimicrobial development needs to be aggressive and target aspects of the pathogens which are least likely to have variable phenotypes. While research along these lines is being done, our failing is perhaps in the pace of it. Research and development for diseases in the poorest countries – those that are most affected by infectious disease – is sorely lacking and innovative mechanisms to provide incentives for the pharmaceutical industry to develop drugs which are in the global public interest need to be devised. Public Private Partnerships (PPPs) have shown some promise but are yet to represent a definitive solution. It is quite remarkable that, until last year, despite 1.5 million people dying of the disease annually, we had failed to produce a single novel TB treatment for 30 years.

Secondly, and perhaps most importantly, mechanisms need to be put in place to ensure responsible antimicrobial usage by clinicians. Overuse, under-use, poor diagnostic techniques and inappropriate choice of antimicrobial account for the bulk of resistant strains emerging world wide. The WHO has taken the lead in this regard with its recommendations under the 'widely and wisely' approach to AMR control.

The overuse of medication is often a response to demanding patients receiving private health care, who want the "latest" and "best" drugs that money can buy. They are unlikely to be satisfied with the use of penicillin as a first line of treatment for their pneumonia and are equally unlikely to be persuaded by an explanation which involves calculating a "greater net social utility".

Antimicrobial effectiveness (AME) is subject to the classical economic scenario of the 'tragedy of the commons'. A 'common' good differs from a public good, in that, although it can be accessed by everyone, it can also be used up, i.e. it is 'rival' in consumption. This is best illustrated with the scenario of a field (or common), to which all the local farmers have access. Before long, the field is over-grazed and of little use to anyone.

It is not just clinicians who are responsible for over grazing the green grass of AME. The use of antimicrobial in the growth of cattle, poultry and fish is contributing to increase in AMR to Enterococci and other microbes which cause infections in humans.

Finally, global health governance strategies need to be continually updated and implemented with a strong emphasis on surveillance. In "Antibiotic resistance as a global threat: Evidence from China, Kuwait and the United States", published this month in *Globalization and Health*, Zhang *et al*. explore the possibility of measuring and monitoring the spread of AMR globally.

In the context of this ongoing erosion of AME and the ominous prospect of returning to an era akin to that of the 'pre-antimicrobial', renewed and innovative efforts to promote new drug development needs to be combined with the coordinated management of 'global common goods consumption' and an ongoing global surveillance strategy.

## Competing interests

The author(s) declares that they have no competing interests

